# The Environmental Occurrence of Pharmaceutical Residues, Agrochemical Contaminants, and Antimicrobial Resistance in a Wastewater-Impacted Urban Water System: A One Health Assessment

**DOI:** 10.3390/molecules31142404

**Published:** 2026-07-08

**Authors:** Amos Misi, Paul Mushonga, Thelma Mari, Greathyl T. Zinyengere, Trinity Njenje, Mary Chipo Mhungu, Pamhidzai Dzomba, Rudo Zhou, Mark F. Zaranyika

**Affiliations:** 1 Chemistry and Earth Sciences Department, University of Zimbabwe, Mount Pleasant, Harare P.O. Box MP167, Zimbabwe; amisi@science.uz.ac.zw (A.M.); pmushonga@science.uz.ac.zw (P.M.); thelma.mari@students.uz.ac.zw (T.M.); greathyl.zinyengere@students.uz.ac.zw (G.T.Z.); trinity.njenje@students.uz.ac.zw (T.N.); maryceemhungu@gmail.com (M.C.M.); 2 Chemistry Department, Bindura University of Science Education, Bindura P. Bag 1020, Zimbabwe; pdzomba@buse.ac.zw (P.D.); rzhou@buse.ac.zw (R.Z.)

**Keywords:** environmental antimicrobial resistance, pharmaceutical occurrence, urban water cycle, wastewater-impacted water systems, One Health, Harare water system, atrazine, herbicides

## Abstract

Urban water security in many cities in the Global South is increasingly challenged by ageing infrastructure and the presence of persistent chemical contaminants. This study investigated the Harare metropolitan water continuum between 2020 and 2024 using a longitudinal, systems-oriented observational framework encompassing wastewater discharge, surface water reservoirs, drinking water treatment, and municipal distribution networks. A three-stage approach was employed, comprising qualitative screening for selected pharmaceuticals at the Lake Chivero water–sediment interface in 2020, spatial assessment of physicochemical stability across the treatment and distribution system in 2021, and targeted qualitative evaluation of pharmaceutical and agrochemical occurrence in wastewater-impacted matrices in 2024. Sulfamethoxazole and trimethoprim were qualitatively identified using high-performance liquid chromatography (HPLC), while atrazine was confirmed by gas chromatography–mass spectrometry (GC–MS). These analyses indicated the continued presence of pharmaceutical and agrochemical residues within wastewater-impacted aquatic compartments associated with the Harare water supply. Physicochemical monitoring revealed elevated ammonia concentrations and reduced free residual chlorine across sections of the distribution network. These conditions coincided with detectable heterotrophic bacterial regrowth at distal consumer endpoints. Phenotypic antimicrobial susceptibility testing of bacterial isolates recovered at the source interface showed limited inhibition responses to sulfamethoxazole and trimethoprim under the experimental conditions used. While the observational nature of this study precludes causal inference, the co-occurrence of chemical residues, physicochemical instability, and bacterial isolates exhibiting reduced inhibition responses highlights conditions of potential relevance for antimicrobial resistance risk within wastewater-influenced urban water systems. These findings underscore the importance of integrated water management strategies addressing wastewater control, source water protection, and distribution system integrity within a One Health context.

## 1. Introduction

Access to safe drinking water is a fundamental human right and a cornerstone of public health, socioeconomic development, and environmental sustainability [1]. Despite advances in water treatment technologies, urban water supply systems in many low- and middle-income countries continue to experience persistent deterioration driven by physicochemical instability, microbial contamination, emerging chemical pollutants, and ageing infrastructure [2]. These challenges are particularly acute in the Global South, where rapid urbanisation and chronic underinvestment have outpaced the maintenance of resilient distribution networks, resulting in declining water quality and system reliability [3].

In Zimbabwe, recurrent outbreaks of waterborne diseases, including cholera and typhoid, have repeatedly exposed systemic vulnerabilities in urban water supply systems, particularly in the Harare metropolitan area [4]. Harare’s municipal water supply is largely dependent on Lake Chivero as its principal raw water source, while the same catchment concurrently receives treated and partially treated wastewater from the metropolitan sewerage system. Urban drainage and effluent from major wastewater treatment plants, particularly the Firle Sewage Treatment Works, enter Lake Chivero primarily through the Mukuvisi River system, establishing a direct hydrological linkage between wastewater discharge and the municipal drinking water supply serving the Harare metropolitan population [5].

**Figure 1:**
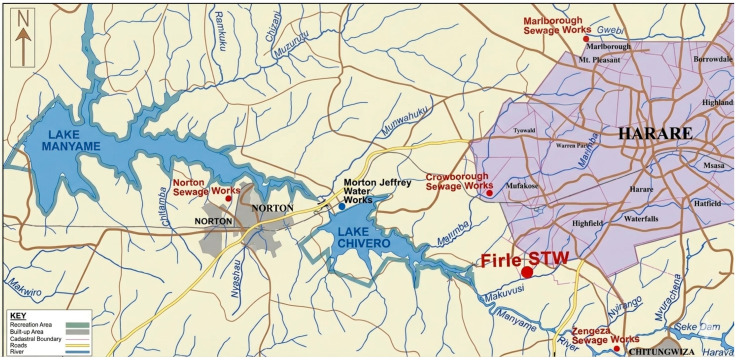
A map of the Harare metropolitan area showing Lake Chivero, major wastewater treatment facilities, and the Morton Jaffray Water Treatment Plant within the Lake Chivero catchment. Adapted from Muserere et al. (2014) [5].

The spatial relationship between wastewater discharge points, Lake Chivero, and the Harare metropolitan water supply system is illustrated in Figure 1.

Consequently, the aquatic environments examined in this study form part of a hydrologically interconnected urban water continuum that ultimately supplies raw water to the municipal treatment system serving the Harare metropolitan population. Waterborne disease outbreaks in the region therefore reflect not only limitations in water and wastewater treatment processes but also risks arising within drinking water distribution networks, where treated water may become re-contaminated due to intermittent supply, pipe leakage, pressure fluctuations, and inadequate residual disinfection [6]. Within this interconnected system, Lake Chivero represents a critical environmental interface where wastewater-derived chemical and microbial inputs may co-occur, with potential implications for downstream physicochemical and microbiological water quality.

Beyond microbial contamination, the increasing prevalence of emerging chemical contaminants has introduced a critical and often underappreciated dimension to urban water quality challenges [7]. In particular, pharmaceutical active compounds (PhACs) are increasingly recognised as contaminants of emerging concern due to their environmental occurrence, biological activity, and documented detection in wastewater effluents and surface waters [8]. Pharmaceuticals enter aquatic systems through incomplete human and veterinary metabolism, improper disposal practices, and the discharge of inadequately treated wastewater [9].

Among these contaminants, antimicrobial compounds have received increasing attention because of their frequent detection in wastewater-impacted aquatic environments and their potential relevance to antimicrobial resistance (AMR) risk assessment. Wastewater treatment plants and impacted receiving waters are widely recognised as environments where microbial communities may be repeatedly exposed to diverse chemical stressors, including residual antibiotics, herbicides, metals, and disinfectants [10,11].

Previous studies have reported associations between such exposure conditions and the occurrence of antimicrobial resistance in environmental microbial communities, without necessarily establishing causality or selection [9,12,13,14,15,16,17,18,19,20,21,22,23,24,25,26,27]. Among the wide range of PhACs detected globally and regionally, sulfamethoxazole (SMX) and trimethoprim (TMP) are of particular relevance to Zimbabwe. These antibiotics are widely used in the country’s public health system, particularly in HIV/AIDS treatment and prophylactic regimens, resulting in high consumption volumes and frequent detection in wastewater and surface waters [28]. Previous studies in Zimbabwe have reported the occurrence of pharmaceutical residues in municipal effluents and receiving waters, indicating incomplete removal during wastewater treatment and continued detection within the aquatic environment [29].

Atrazine was included in this study as a representative non-antibiotic agrochemical widely used in maize production systems in Zimbabwe and the surrounding agricultural catchments. Its inclusion provides contextual insight into mixed chemical pressures that may occur in wastewater-influenced urban water systems rather than direct evidence of co-selection processes [30,31].

Conventional wastewater treatment processes consistently achieve only partial removal of many pharmaceutical and agrochemical compounds, allowing for their continued presence in aquatic environments [32]. While such detections have been discussed in the literature in relation to AMR risk, the specific mechanisms linking chemical occurrence and microbial resistance remain complex and context-dependent, particularly in observational environmental studies [33].

While substantial progress has been made in characterising individual dimensions of water contamination, integrated assessments that jointly examine chemical occurrence, physicochemical instability, and microbial indicators across interconnected urban water systems remain limited, particularly in African urban contexts [34]. In Zimbabwe, available evidence is largely confined to routine physicochemical monitoring or isolated microbiological investigations, with limited empirical integration of chemical exposure and microbial response across the wastewater–source water–distribution continuum [35].

To address these gaps, the present study adopts a sequential, systems-based three-stage longitudinal framework that treats the Harare urban water cycle as an interconnected environmental continuum linking wastewater discharge, surface water reservoirs, drinking water treatment infrastructure, and municipal distribution networks. Within this framework, Lake Chivero functions simultaneously as a wastewater-impacted receiving water body and the primary raw water source for Harare, establishing a shared chemical–microbial interface within the metropolitan water cycle [36]. Rather than examining chemical contaminants or microbial indicators in isolation, this study integrates qualitative pharmaceutical screening, physicochemical monitoring, and phenotypic antimicrobial susceptibility testing to examine their environmental co-occurrence across multiple compartments of the urban water supply system.

Accordingly, the objective of this study was to investigate the environmental occurrence and co-distribution of selected pharmaceutical contaminants and their relationship with antimicrobial resistance indicators within the Harare urban water continuum. Specifically, the study aimed to (i) detect the occurrence of the antibiotics sulfamethoxazole and trimethoprim in wastewater-impacted aquatic environments associated with Lake Chivero, (ii) evaluate physicochemical and microbiological water quality dynamics across the urban water supply system, and (iii) examine the environmental coexistence of pharmaceutical residues, agrochemical stressors, and bacterial isolates exhibiting phenotypic inhibition responses under defined assay conditions within this interconnected water system.

Framed within a One Health perspective, this work provides an integrated baseline describing chemical and microbial co-occurrence within an urban water system of public health relevance, supporting future risk assessment and water safety management strategies in the Harare metropolitan region.

## 2. Results

### 2.1. Chemical Stressors at the Lake Chivero Source Interface

#### 2.1.1. Baseline Evidence of Antibiotic Presence (2020 Screening)

**Figure 2:**
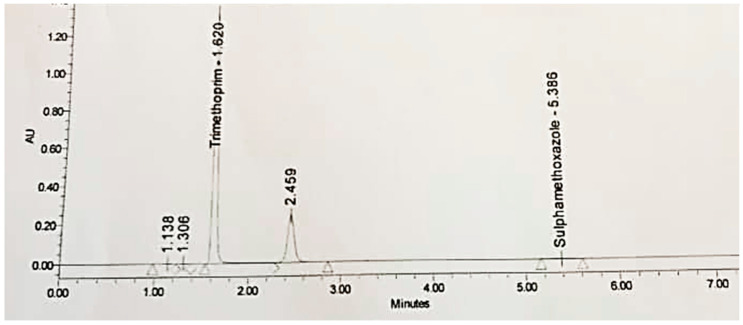
Chromatograms obtained from the analysis of Lake Chivero’s water.

Chromatographic screening conducted in 2020 confirmed the presence of selected antibiotics in wastewater-impacted samples collected at the Lake Chivero source interface. Following ultrasonic-assisted matrix solid-phase dispersion (MSPD) extraction and HPLC analysis [37], discrete and reproducible chromatographic peaks corresponding to trimethoprim (TMP) and sulfamethoxazole (SMX) were observed, as shown in Figure 2.

TMP eluted at approximately 1.620 min, while SMX eluted at approximately 5.386 min. These retention times matched those of analytical standards analysed under the same chromatographic conditions. Peak signals for both compounds were consistently observed across replicate injections, confirming their occurrence in the analysed wastewater matrices.

In addition to the identified TMP and SMX peaks, minor unresolved peaks were present in the chromatograms (Figure 2). These peaks were not assigned to specific compounds due to the absence of corresponding reference standards and were therefore not interpreted further. Their presence indicates additional chromatographic features within the wastewater matrix that were not characterised in the present work.

This 2020 dataset was limited to qualitative detection. Concentrations were not calculated, and no quantitative comparison with subsequent datasets was undertaken due to differences in extraction methodology and analytical configuration. No external calibration curves or absolute concentration calculations were performed. Chromatographic responses were interpreted qualitatively based on retention time agreement with standards and peak reproducibility.

#### 2.1.2. GC–MS Identification of Organic Micropollutants

During the 2024 phase of the study, GC–MS analysis was applied to evaluate the occurrence of non-polar organic micropollutants in wastewater effluent collected from the Firle Wastewater Treatment Plant, which is hydrologically linked to Lake Chivero through the Mukuvisi River system. An ethyl acetate–acetonitrile mixture was used to obtain extracts suitable for GC–MS analysis of complex wastewater matrices.

GC–MS analysis identified the herbicide atrazine in wastewater samples. Atrazine was detected at a retention time of 23.08 min, with identification supported by a spectral similarity score of 96.1 against reference library spectra. The corresponding mass spectrum exhibited diagnostic fragment ions at *m*/*z* 200.1, 215.1, 173.1, 58.1, and 43.1, consistent with atrazine fragmentation patterns reported in the literature [38,39,40]. Extracted ion chromatograms further supported the assignment.

The atrazine chromatographic signal produced a peak area of 1,955,026 under the analytical conditions used. Because the study did not include calibration curves, internal standards, or quantitative response normalisation, peak areas are reported descriptively and are not interpreted as concentrations or absolute abundance.

The qualitative identification of atrazine alongside TMP and SMX provides evidence of co-occurring agrochemical and pharmaceutical residues in wastewater impacted matrices at the Lake Chivero source interface.

**Figure 3:**
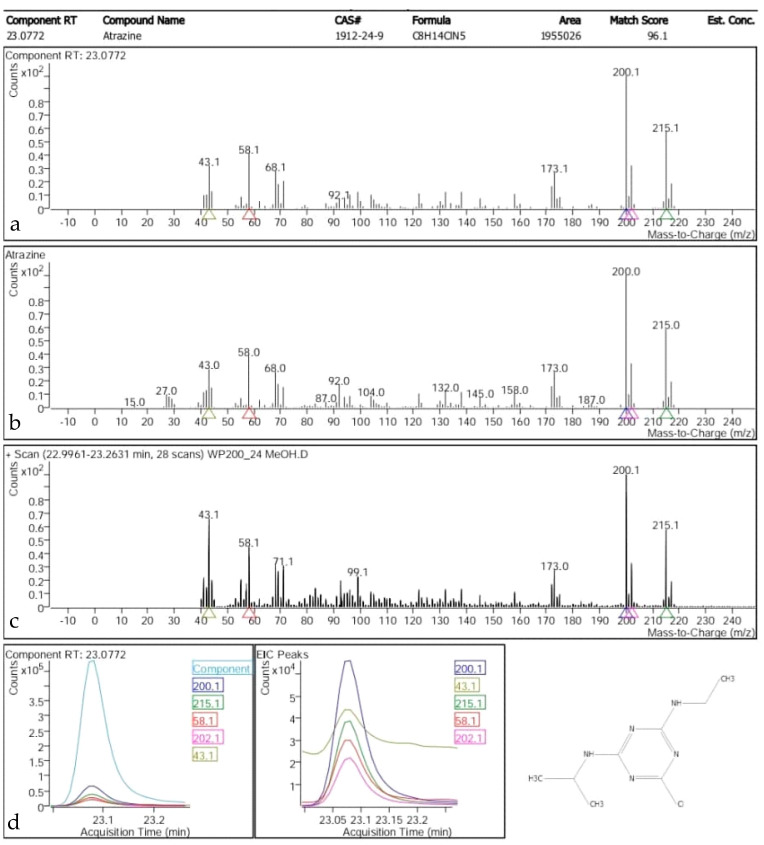
GC–MS identification of atrazine in wastewater effluent from the Firle Wastewater Treatment Plant. (**a**) Library-matched mass spectrum of the detected compound showing diagnostic fragment ions and a spectral match score of 96.1 for atrazine (CAS 1912-24-9). (**b**) Reference mass spectrum of atrazine obtained from the spectral database for comparison. (**c**) Experimental mass spectrum obtained from the wastewater extract, showing characteristic fragment ions consistent with atrazine fragmentation. (**d**) Extracted ion chromatograms (EICs) of diagnostic ions (*m*/*z* 200, 215, 173, 58, and 43) demonstrating co-elution at a retention time of approximately 23.08 min, together with the molecular structure of atrazine.

Figure 3 presents the GC–MS chromatogram and associated mass spectrum for atrazine used for confirmation.

### 2.2. Physicochemical Instability Across the Harare Urban Water System Continuum (2021)

#### 2.2.1. Physicochemical Context: Water Stability Across the Urban Water Cycle

Physicochemical water quality across the Harare urban water system exhibited spatial variability from raw source water through treatment infrastructure to distribution network endpoints (Table 1). Variations in pH, turbidity, colour, ammonia, conductivity, and trace metals reflect differences observed across sampling locations along the supply continuum.

#### 2.2.2. Harare Physicochemical Baseline and Operational Trends (2021)

**Figure 4:**
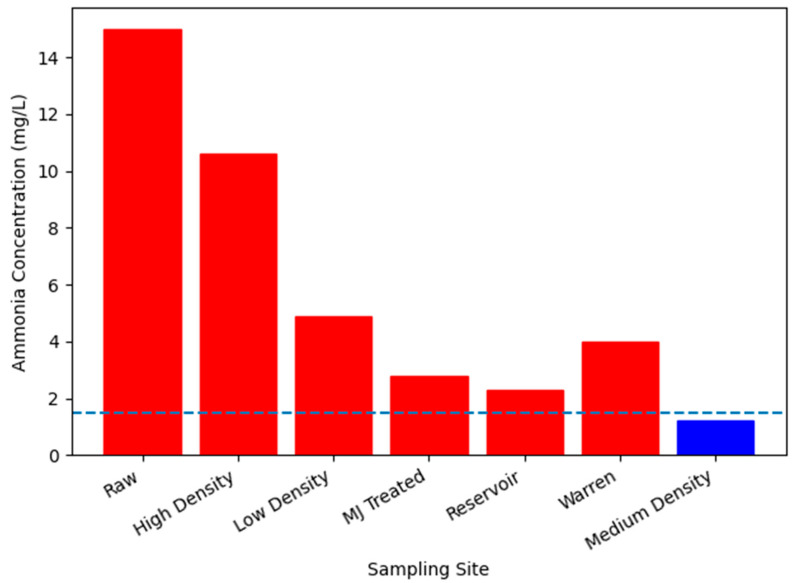
Mean ammonia concentrations at Harare sampling sites relative to the WHO reference value.

During the July–September 2021 assessment, one-way analysis of variance (ANOVA) indicated statistically significant spatial variability for pH (*p* < 0.001). Ammonia–nitrogen also differed significantly by sampling location (*p* < 0.05), with the highest mean ammonia concentration recorded in raw water (15 ± 3.86 mg L^−1^). Across site categories, ammonia concentrations exceeded the WHO reference value of 1.5 mg L^−1^ in 6 of 7 site categories (85.7%), based on the dataset summarised in Table 1 and Figure 4.

Free residual chlorine concentrations were lowest at distribution system reservoirs and selected downstream sites compared with the treatment plant outlet (Table 2). The mean free residual chlorine at the treatment plant outlet (0.30 ± 0.17 mg L^−1^) declined to approximately 0.02 mg L^−1^ in service reservoirs. Total residual chlorine values exhibited a different spatial pattern from free residual chlorine, with divergence between free and total residual chlorine observed across sites (Table 2). Because this study is observational, these spatial patterns are reported as measured without attributing causality to any single driver.

Iron and aluminium concentrations decreased substantially between raw water and the Morton Jaffray treatment plant outlet (*p* < 0.001). Downstream variability in iron and aluminium was not statistically significant (*p* > 0.05), across the distribution sites. Aluminium reached 0.43 ± 0.10 mg L^−1^ in high-density residential areas compared with 0.10 ± 0.08 mg L^−1^ at the treatment outlet (Table 1), indicating site-dependent variation within the distribution system.

### 2.3. Microbial Recovery Within the Harare Distribution Network (2021)

#### 2.3.1. Comparison of Microbial Contamination Levels Across Sampling Sites

Microbiological assessment during the July–September 2021 campaign showed spatial variation in heterotrophic plate counts (HPC) across the source, treatment, and distribution network. ANOVA indicated that sampling location had a statistically significant effect on HPC (*p* < 0.05).

**Figure 5:**
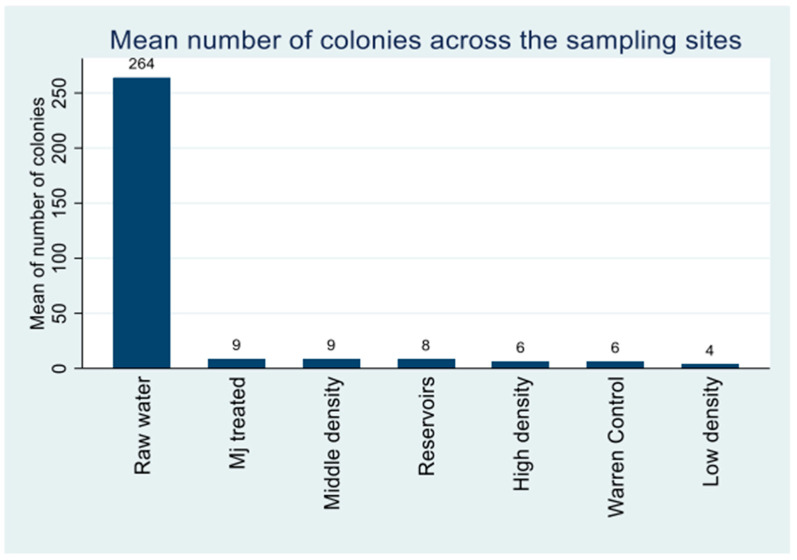
Mean heterotrophic plate counts (CFU mL^−1^) across raw water, treated water, and distribution sampling sites (2021).

Raw water samples from Lake Chivero had the highest HPC values (264 ± 49.4 CFU mL^−1^; Figure 5). Following treatment at the Morton Jaffray Water Treatment Plant, HPC values decreased markedly (approximately 9 CFU mL^−1^), indicating reduced heterotrophic bacterial abundance at the point of treatment.

Detectable HPC values were observed again at downstream distribution sampling sites. Although all reported HPC values remained below the WHO reference level of 500 CFU mL^−1^, the increase relative to the treatment outlet indicates changes in distribution system microbiological conditions at sampled endpoints. This downstream increase occurred alongside low free residual chlorine values at distribution sites (Table 2), and the two observations are reported here as co-occurring patterns in the dataset rather than as cause-and-effect.

#### 2.3.2. Identification of Indicator and Potentially Pathogenic Bacteria

Selective and differential culturing performed during the 2021 campaign identified multiple taxa of public health relevance, including *Escherichia coli*, *Shigella* spp., *Salmonella* spp., *Klebsiella* spp., and *Proteus* spp. (Table 3) [41]. These organisms are recognised as indicators of faecal contamination and/or as potential waterborne pathogens.

All five taxa were detected in raw water samples from Lake Chivero (Table 4). None were detected at the outlet of the Morton Jaffray Water Treatment Plant, consistent with removal at the treatment stage in the sampled events.

Within the distribution network, *E. coli* was detected at medium- and high-density residential sampling points, while *Salmonella* spp. were detected at distal high-density endpoints (Table 4). This pattern absence at the treatment outlet with detection at some points of use indicates site-specific microbiological findings within the distribution system during the sampling period.

#### 2.3.3. Public Health Relevance of Identified Bacteria (2021)

The taxa detected during the 2021 assessment differ in persistence characteristics and public health relevance. *E. coli* and *Salmonella* spp. are notable because of their recognised role as indicators of faecal contamination and their association with waterborne disease outbreaks.

The intermittent detection of *E. coli* and *Salmonella* spp. at consumer endpoints alongside their absence at the treatment outlets in the sampled events, indicates that microbiological conditions differed across the distribution system during the study period.

These observations support the interpretation that distribution system conditions can influence microbiological quality at points of use, while the present dataset does not establish the source, direction, or mechanism of contamination.

### 2.4. Antibiotic Susceptibility of Environmentally Relevant Bacterial Isolates

Inhibition zone diameters produced by trimethoprim (TMP) and sulfamethoxazole (SMX) against environmental isolates are summarised in Table 5. Phenotypic inhibition testing showed species-specific responses under the assay conditions used.

*Shigella* spp. exhibited inhibition zones against TMP across the tested concentration range (10–1.25 mg mL^−1^), with mean inhibition diameters of 32.00 ± 1.73 mm (10 mg mL^−1^), 22.00 ± 1.00 mm (5 mg mL^−1^), 20.33 ± 0.58 mm (2.5 mg mL^−1^), and 22.00 ± 0.00 mm (1.25 mg mL^−1^). In contrast, no inhibition zones were observed for SMX under the tested concentrations for *Shigella* spp.

For *E. coli* and *Salmonella* spp., no inhibition zones were observed for TMP or SMX across the tested concentrations. These results are reported as an absence of inhibition under the experimental conditions employed and are not interpreted here using clinical breakpoints or MIC-based resistance categories.

### 2.5. Qualitative Chemical Occurrence Across the Urban Water System (2024)

#### Occurrence of Sulfamethoxazole and Trimethoprim in Firle Wastewater Effluent (2024)

The 2024 phase applied an optimised liquid–liquid extraction (LLE) workflow using an ethyl acetate–acetonitrile mixture to obtain extracts suitable for HPLC screening in matrix-rich wastewater-impacted samples.

**Figure 6:**
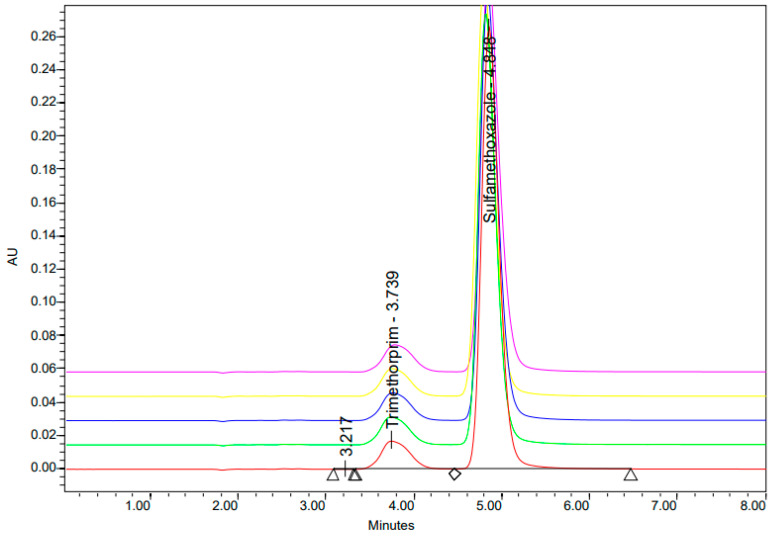
Chromatograms of sulfamethoxazole and trimethoprim standards.

HPLC analysis of SMX–TMP analytical standards yielded peaks at retention times of 4.848 min (SMX) and 3.739 min (TMP) (Figure 6). No absolute concentration calculations were performed. Chromatographic responses were therefore interpreted qualitatively using retention time agreement and peak reproducibility to support analyte identification.

**Figure 7:**
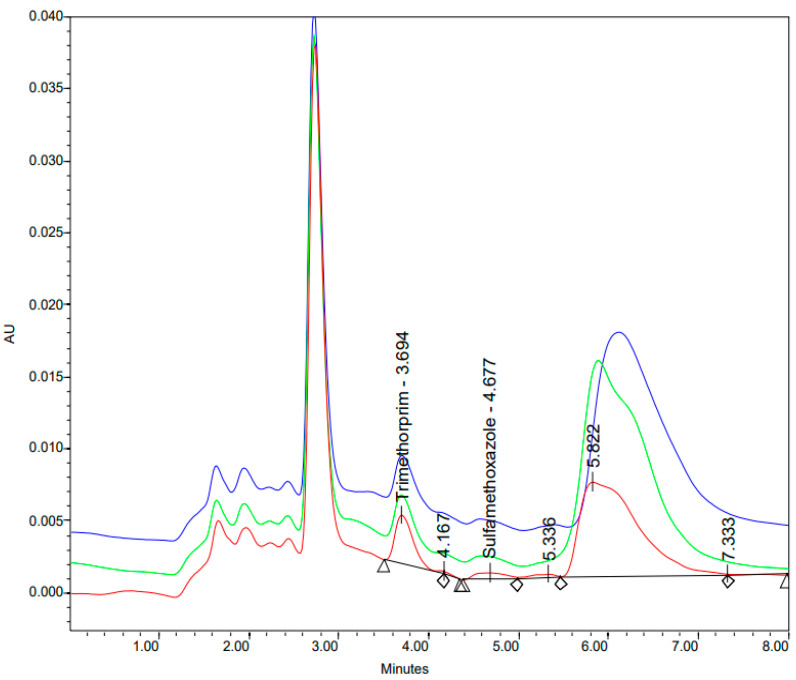
Representative HPLC chromatograms of LLE environmental extracts collected at the wastewater treatment plant outlet (2024), showing peaks consistent with sulfamethoxazole (SMX) and trimethoprim (TMP) based on retention time agreement with analytical standards.

In the environmental extracts (Figure 7), analyte identification was based on reproducible peak resolution and retention time matching. Minor shifts in retention time were observed in some wastewater-impacted chromatograms; however, observed peaks remained within the acceptable window used for qualitative identification under the chromatographic conditions applied.

Additional peaks were also present in environmental chromatograms, indicating other chromatographically detectable constituents within the sampled matrices; these peaks were not assigned to specific compounds.

## 3. Discussion

### 3.1. Analytical Considerations, Identification Confidence, and Study Constraints

Environmental monitoring of pharmaceuticals and agrochemicals in wastewater-impacted systems is challenged by complex matrices that can affect extraction efficiency, chromatographic behaviour, and signal interpretation. In this study, the analytical workflow was implemented across temporally distinct stages that employed different extraction configurations designed to obtain interpretable chromatographic data in matrix-rich samples. Because the study scope prioritised qualitative identification rather than absolute quantification, compound detection was interpreted using retention time agreement with analytical standards (for SMX/TMP) and mass spectral library matching with diagnostic ions (for atrazine).

A key limitation is that no external calibration curves, internal standards, or concentration calculations were performed for the HPLC datasets, and GC–MS confirmation of atrazine relied on reference library matching in the absence of certified atrazine standards. Accordingly, findings are most appropriately interpreted as evidence of occurrence and co-occurrence across wastewater-impacted compartments rather than evidence of concentration trends, attenuation performance, or exposure magnitude.

Where replicate injections yielded stable chromatographic signals (reported as low injection-level variability), this supports instrumental repeatability under the applied conditions, but it does not by itself demonstrate environmental persistence, steady-state loading, or temporal comparability across campaigns that used different extraction configurations. The discussion below therefore focuses on patterns that are directly supported by the dataset: qualitative detection of target compounds, observed physicochemical instability across sites, microbial regrowth indicators, and phenotypic inhibition response patterns measured under the assay conditions used.

### 3.2. Co-Occurrence of Pharmaceuticals and Agrochemical in Wastewater-Impacted Matrices

Qualitative HPLC screening identified sulfamethoxazole (SMX) and trimethoprim (TMP) in wastewater-impacted water collected at the Lake Chivero source interface during the 2020 baseline screening, based on retention time agreement with analytical standards and peak reproducibility. In a later monitoring phase (2024), the same pharmaceuticals were qualitatively detected in wastewater effluent collected from the Firle Wastewater Treatment Plant, which is hydrologically linked to Lake Chivero through the Mukuvisi River system. Although the study design and analytical configuration do not support concentration estimates or direct temporal quantification, these observations indicate that SMX and TMP occur within multiple compartments of the wastewater-influenced urban water system examined in this work.

GC–MS analysis further identified atrazine in wastewater effluent collected from the Firle Wastewater Treatment Plant, supported by a high library similarity score and diagnostic fragment ions.

Together, these findings indicate that pharmaceutical residues (SMX/TMP) and a representative non-antibiotic contaminant (atrazine) can occur within wastewater-impacted matrices associated with the Harare water continuum. Such co-occurrence is environmentally relevant because aquatic compartments receiving wastewater inputs frequently contain mixtures of biologically active chemicals. In addition to antibiotics, certain herbicides have been discussed in the literature as non-antibiotic stressors capable of influencing bacterial responses to antimicrobial compounds [42,43,44,45,46,47]. Experimental studies have shown that exposure to atrazine can alter antimicrobial susceptibility profiles in environmental isolates of Pseudomonas aeruginosa; however, these changes were not associated with increased expression of the MexAB–OprM efflux pump, suggesting the involvement of alternative cellular mechanisms. More broadly, non-antibiotic stressors such as pesticides have been reported to influence bacterial responses to antibiotics through multiple pathways, including reduced outer membrane permeability, modulation of efflux systems, stress-induced genetic variation, and enhanced horizontal gene transfer processes that facilitate the dissemination of antimicrobial resistance genes.

Within this context, the coexistence of atrazine with SMX and TMP within the same wastewater-influenced system highlights conditions under which multiple chemical stressors may interact with environmental microbial communities and potentially influence both resistance acquisition and dissemination. However, the present dataset does not establish causal relationships between chemical occurrence and microbial outcomes, nor does it quantify exposure levels required to infer resistance selection mechanisms.

### 3.3. Physicochemical Instability and Disinfectant Residual Loss Across the Distribution System

The 2021 monitoring dataset demonstrates that physicochemical parameters varied across the Harare water continuum, including elevated ammonia in raw water and low free residual chlorine values at distribution system sites relative to the treatment plant outlet. Raw water ammonia was reported at 15 ± 3.86 mg L^−1^, and mean free residual chlorine declined from 0.30 ± 0.17 mg L^−1^ at the plant outlet to approximately 0.02 ± 0.03 mg L^−1^ at reservoirs.

These observed patterns indicate reduced disinfectant residual persistence at downstream points during the sampling period. Because this is an observational study, the data support reporting this decline as a spatial pattern without attributing it to a single driver in the absence of supporting mass-balance or kinetic modelling. Nonetheless, reduced free residual chlorine at distal sites is operationally consistent with conditions under which microbial regrowth and distribution system vulnerability can occur, particularly in systems subject to variable residence time and intermittent supply conditions.

### 3.4. Microbial Regrowth Indicators and Distribution System Microbiological Findings

Microbiological monitoring in 2021 showed significant spatial variation in heterotrophic plate counts (HPCs), with raw water values of 264 ± 49.4 CFU mL^−1^, marked reduction at the treatment outlet (approximately 9 CFU mL^−1^), and detectable HPC values again at downstream distribution sampling sites. These downstream observations occurred alongside low free residual chlorine values at distribution sites, and the manuscript appropriately treats these as co-occurring patterns rather than definitive evidence of causality.

Selective culturing identified taxa of public health relevance (including *E. coli* and *Salmonella* spp.) in raw water and at selected consumer endpoints, with absence at the treatment outlet in the sampled events. This pattern supports the interpretation that microbiological conditions can differ across the distribution system, and that distribution-system performance can influence microbiological quality at points of use, while the dataset does not establish the specific source, direction, or mechanism of contamination.

### 3.5. Phenotypic Inhibition Response Patterns and Relevance to AMR Risk Discussions

Phenotypic inhibition testing performed on isolates from the Lake Chivero interface demonstrated species-specific responses under the assay conditions used. *Shigella* spp. exhibited inhibition zones to TMP across the tested concentration range, while SMX produced no inhibition zones for *Shigella* spp. under the tested conditions. For *E. coli* and *Salmonella* spp., no inhibition zones were observed for either TMP or SMX across the tested concentrations, and the manuscript appropriately reports these as absence of inhibition under assay conditions rather than assigning clinical breakpoint-based resistance categories.

Given the observational design and qualitative chemical occurrence data, the most defensible interpretation is that chemical residues (SMX/TMP; atrazine) and bacterial isolates exhibiting limited inhibition responses were detected within the same wastewater-impacted system compartments, indicating co-occurrence of bioactive chemicals and phenotypic response patterns relevant to antimicrobial resistance risk discussions. This does not demonstrate selection, horizontal gene transfer, or persistence of resistance determinants over time. It does, however, provide an integrated baseline describing the intersection of (i) wastewater-associated chemical occurrence, (ii) distribution system physicochemical instability, and (iii) microbial regrowth indicators within the Harare water continuum.

### 3.6. Integration with Prior Zimbabwean Evidence and Study Limitations

Previous studies in Zimbabwe have reported antimicrobial resistance genes (e.g., sulfonamide resistance determinants) and multidrug-resistant organisms in wastewater and receiving waters. These reports provide important context suggesting that wastewater-influenced aquatic environments in the region can contain genetic reservoirs of resistance. However, these external findings cannot be treated as genotypic confirmation of the isolates tested in the present study, because resistance genes were not measured here. The present results therefore stand as phenotypic inhibition response evidence, complemented by literature-based context rather than direct genetic linkage [10,48,49].

The major limitations are as follows: (i) qualitative chemical identification without calibration-based concentrations; (ii) a lack of certified atrazine standards for GC–MS confirmation and quantification; (iii) phenotypic susceptibility testing restricted to isolates from the source interface rather than distal endpoints; and (iv) the absence of molecular characterisation (PCR/metagenomics) to identify resistance determinants and mobile genetic elements. These limitations define the appropriate scope of inference: the study supports occurrence/co-occurrence and distribution system vulnerability patterns, not mechanistic claims of selection, evolution, or dissemination pathways.

### 3.7. Implications and Future Work

From a water safety management perspective, the observed decline in disinfectant residuals across parts of the distribution system, together with microbial regrowth indicators and the detection of wastewater-associated chemical residues, supports the need for integrated interventions spanning wastewater control, source water protection, and distribution system integrity. Future work should prioritise (i) quantitative chemical analysis with internal standards and method detection limits; (ii) targeted molecular detection of resistance genes and mobile genetic elements to link phenotype to genotype; and (iii) biofilm-focused sampling within distribution infrastructure to evaluate microbial reservoirs under low disinfectant residual conditions.

## 4. Materials and Methods

### 4.1. Study Design and Temporal Framework

This study used a sequential, three-stage observational framework (2020–2024) to examine chemical occurrence, physicochemical variability, and microbiological indicators across the Harare urban water continuum, including Firle wastewater effluent, the Lake Chivero source interface, drinking water treatment, and downstream distribution endpoints. The design integrated temporally separated datasets to provide an occurrence-based baseline and a systems-level description of co-occurring chemical and microbiological patterns across key compartments of the urban water cycle.

Because the study employed qualitative chemical identification and phenotypic microbiology assays, it was designed to describe co-occurrence patterns and distribution system vulnerability indicators, rather than to establish causal mechanisms, quantify exposure magnitudes, or infer evolutionary processes.

### 4.2. Longitudinal Integration and Taxonomic Continuity

The research was structured around three temporally distinct stages to support consistent mapping of the chemical–microbial interface across the Harare urban water continuum.

**Stage I: Preliminary Chemical Baseline (2020).**

Initial screening established the foundational chemical pressure within the Lake Chivero water–sediment interface. Using a solid-phase extraction approach, this stage confirmed the chronic presence of a binary antibiotic mixture comprising sulfamethoxazole and trimethoprim, providing the baseline against which subsequent biological responses were evaluated.

**Stage II: Chronic Exposure and Taxonomic Resolution (2021).**

A high-resolution spatial assessment was conducted across raw water sources and multiple points within the distribution network. This stage resolved the taxonomic composition of the resident microbial community, with targeted isolation and characterisation of *Escherichia coli* and *Salmonella* species. 

**Stage III: Ecological Stress and AMR Validation (2024).**

A targeted qualitative analytical workflow was applied to evaluate the occurrence of sulfamethoxazole and trimethoprim in wastewater effluent samples collected at the Firle Sewage Treatment Plant outlet discharging toward Lake Chivero via the Mukuvisi River. Atrazine presence was assessed by GC–MS library matching. In parallel, representative bacterial isolates recovered from the Lake Chivero interface were subjected to phenotypic inhibition response testing against sulfamethoxazole and trimethoprim under defined assay conditions.

### 4.3. Sampling Campaigns and Sampling Framework

To capture spatial variability and distribution system integrity, the sampling process was structured into two coordinated longitudinal phases:

**Synchronized Primary Assessment (July–September 2021)**

A spatially distributed sampling strategy was implemented across the Harare urban water continuum, encompassing raw source water, treated drinking water, storage reservoirs, and distribution system endpoints. Sampling locations included Lake Chivero source interface water, treated water at the Morton Jaffray Water Treatment Plant outlet, and three service reservoirs (Highlands, Lochinvar, and Greendale). Additional samples were collected from a designated control site (Warren) and from distribution points located within low-, medium-, and high-density residential areas, selected as representative consumer endpoints.

Sampling was conducted over a three-month period (July–September 2021), during which a total of 42 water samples were collected. Each sampling location was visited monthly, with duplicate samples collected per visit, resulting in repeated measurements across the study period. Where applicable, physicochemical and microbiological analyses were performed in triplicate to ensure measurement repeatability. Reported values are presented as site-specific means with corresponding standard deviations derived from repeated measurements.

**Acute monitoring and qualitative validation (January–July 2024)**

The 2024 phase focused on qualitative identification of selected pharmaceutical and agrochemical contaminants in wastewater-impacted matrices entering the Lake Chivero basin from the wastewater treatment interface. In parallel, representative bacterial isolates recovered at the Lake Chivero interface were subjected to phenotypic inhibition response testing to characterise species-specific responses to sulfamethoxazole and trimethoprim under defined assay conditions.

### 4.4. Sample Collection, Containers, Preservation, and Handling

Standardised sampling and preservation procedures were applied across all study sites to ensure methodological consistency and data comparability.

#### 4.4.1. Container Preparation

Single-use 100 mL sampling containers (Pro-Plastics, Harare, Zimbabwe) were pre-cleaned with hot water and chemically disinfected by immersion in 1% sodium hypochlorite for 30 min, followed by thorough rinsing with sterile distilled water and air drying at room temperature.

#### 4.4.2. Drinking Water Sampling

At taps and reservoirs, samples were collected following a standardised flushing protocol. Taps were flushed at maximum flow for 2 min to remove stagnant water, then sampled at normal flow. Sterile glass bottles pre-dosed with sodium thiosulfate were used at chlorinated locations to neutralise residual disinfectant and preserve microbial integrity.

Prior to sampling, taps were disinfected by swabbing internal and external surfaces with sodium hypochlorite solution and allowing sufficient contact time to minimise external contamination. Water was then allowed to flow at a constant rate prior to sample collection to reduce disturbance of pipe biofilms.

#### 4.4.3. Lake Chivero Interface Sampling (Water Only)

Within Lake Chivero, sampling targeted the sediment–water interface zone by collecting aqueous-phase water immediately above the sediment surface. No sediment material was collected or analysed. Sampling was conducted at three lacustrine nodes (Upper, Middle, Lower Lake Chivero) selected to represent gradients in hydraulic residence time and proximity to treated effluent discharge points.

A weighted sterile horizontal Van Dorn sampler (or a modified suction device where deployment was constrained) was used to withdraw water from the 0–10 cm zone above the sediment bed. Five discrete 1 L subsamples were collected per node within a defined radius and pooled to generate a 5 L composite sample used for chemical analysis. Physicochemical and microbiological measurements were conducted in analytical triplicate where applicable. All lake water samples were collected in pre-cleaned amber glass containers, stored on ice, and transported under dark conditions to minimise photodegradation prior to extraction.

#### 4.4.4. Treated Wastewater Effluent Sampling

Treated wastewater effluent samples were collected from the final effluent stream immediately prior to discharge into the receiving system to characterise residual chemical loading entering the lake catchment. Grab samples were collected under steady-flow conditions into pre-cleaned 5 L containers, immediately chilled, and transported for laboratory analysis. Effluent samples were processed using a matrix-adapted extraction protocol distinct from that applied to lake water samples to accommodate higher organic complexity and ionic strength.

#### 4.4.5. Quality Assurance and Reproducibility

All sampling equipment was field-rinsed with site water prior to collection and solvent-rinsed between sites to prevent cross-contamination. Field blanks, transport blanks, and duplicate samples were included at predefined intervals. Replicate sampling at selected nodes was conducted to verify procedural consistency.

All samples were transported on ice (≈4 °C) and analysed within recommended holding times.

### 4.5. Physicochemical Water Quality Analysis

In situ measurements of *pH*, temperature, and electrical conductivity (EC) were conducted at the point of collection using calibrated portable instruments (pH: Hanna HI98107 manufactured by HANNA instruments Inc, Woonsocket-USA; EC: Hach CDC401 manufactured by Hach Company Colorado-USA). Free and total residual chlorine were determined immediately after sampling using the DPD colorimetric method (Standard Methods 4500-Cl G) [50].

All laboratory analyses were conducted at the Analytical Chemistry Laboratory, University of Zimbabwe.

Physicochemical water quality analyses were conducted at both the Harare City Council Water Laboratories and the Analytical Chemistry Laboratory, University of Zimbabwe, following Standard Methods for the Examination of Water and Wastewater. High-performance liquid chromatography (HPLC) analyses for pharmaceutical identification were performed at Pharmanova Laboratories (Harare, Zimbabwe). Gas chromatography–mass spectrometry (GC–MS) analyses for agrochemical identification were conducted at the Government Analyst Laboratories (Zimbabwe). Turbidity was measured using a Hach 2100Q turbidimeter (Loveland, CO, USA). Ammonia–nitrogen was determined using the Nessler method. Iron and aluminium were analysed by using flame atomic absorption spectrophotometry (Shimadzu AA-7000 manufactured by Shimadzu, Kyoto, Japan) following EPA Method 200.7 [51]. All laboratories applied their respective internal quality assurance and instrument calibration procedures appropriate to the analytical methods employed.

### 4.6. Microbiological Procedures

Heterotrophic plate counts (HPCs) were determined using the pour plate technique on nutrient agar. Plates were incubated aerobically at 22 °C and 37 °C for 24–120 h to capture both environmental and slow growing heterotrophic bacteria.

Where bacterial loads were high, particularly for lake water samples, serial ten-fold dilutions were prepared prior to plating to ensure countable colony ranges (30–300 CFU).

Colony counts were converted to CFU mL^−1^ for reporting consistency.

Faecal indicator and opportunistic pathogenic bacteria (*Escherichia coli*, *Salmonella* spp., *Shigella* spp., *Klebsiella* spp., and *Proteus* spp.) were isolated using selective and differential media, followed by Gram staining and biochemical confirmation using Triple Sugar Iron (TSI) tests.

### 4.7. Antibiotic Susceptibility Testing and Environmental AMR Assessment

Environmental bacterial isolates were standardised to a 0.5 McFarland turbidity standard (approximately 1 × 10^8^ CFU mL^−1^) for inoculum normalisation. Suspensions were prepared by transferring colonies into sterile 0.85% saline and adjusting turbidity to the McFarland standard.

Phenotypic inhibition responses to trimethoprim (TMP) and sulfamethoxazole (SMX) were evaluated using an agar well diffusion assay on Mueller–Hinton agar (HiMedia Laboratories Pvt. Ltd., Mumbai, India). Plates were inoculated with standardised suspensions using sterile swabs to obtain uniform lawns. Wells (~5 mm diameter) were aseptically punched, and 50 µL of antibiotic solution was added to each well. TMP and SMX solutions were prepared in sterile distilled water and applied at 1.25, 2.5, 5.0, and 10 mg mL^−1^. All assays were performed in triplicate and incubated aerobically at 37 °C for 24 h prior to measurement of inhibition zones.

Because agar well diffusion is not a CLSI-standard susceptibility method and no clinical breakpoints were applied, the results are reported as inhibition zone diameters (or absence of inhibition) under the assay conditions used, rather than as clinical susceptibility categories. Quality control procedures were applied with respect to media preparation, inoculum standardisation, incubation conditions, and assay reproducibility, and solvent controls were included as applicable. The results were interpreted as comparative phenotypic inhibition response patterns relevant to the study context.

### 4.8. Organic Extraction of Organic from Lake Water and Treated Wastewater Effluent

To account for marked differences in matrix composition between lake water and treated wastewater effluent, two extraction procedures were applied.

#### 4.8.1. Lake Water Samples (Ultrasonic-Assisted Dispersive Solid-Phase Extraction)

Organic residues in lake water samples were extracted using an ultrasonic-assisted dispersive solid-phase extraction procedure adapted from previously validated methods [37]. One litre of sample was transferred into a separating funnel and vigorously shaken with ten millilitres of acetonitrile. Disodium EDTA (0.1 M; 5 mL) and McIlvaine buffer (pH 4; 10 mL) were added to chelate metal ions and promote release of complexed analytes.

Magnesium sulphate and sodium chloride (0.5 g each) were added to facilitate salting-out and phase separation. The mixture was centrifuged at 3000 rpm for 10 min, and the organic supernatant was transferred. Hydrophilic–lipophilic balance (HLB) sorbent (40 mg) was added. The suspension was ultrasonicated for 15 min and centrifuged again. The sorbent phase was collected and packed into a 6 mL polypropylene syringe barrel, washed with ultrapure water, and vacuum-dried for 2 h. Elution was performed with 12 mL methanol. The eluate was evaporated to near dryness under reduced pressure and reconstituted in 500 µL methanol. Extracts were filtered through 0.22 µm glass fibre filters, transferred to amber vials, and stored refrigerated prior to analysis.

#### 4.8.2. Treated Wastewater Effluent (Liquid–Liquid Extraction)

Treated wastewater effluent samples were extracted using an optimised liquid–liquid extraction protocol adapted from Zafar et al. [52], with modifications for matrix-rich effluent. Five hundred millilitres of effluent were acidified to approximately pH 2.5 to suppress ionisation of weak acids and enhance partitioning.

Extraction employed a mixed solvent system of ethyl acetate and acetonitrile. A 1:1 (*v*/*v*) ethyl acetate/acetonitrile ratio was selected based on improved chromatographic response and peak resolution under the study conditions. The solvent mixture was applied by vigorous shaking, followed by phase separation and recovery of the organic layer. Extracts were concentrated under controlled conditions and reconstituted in an appropriate solvent prior to analysis.

#### 4.8.3. Instrumental Analysis and Compound Confirmation

Qualitative chromatographic analysis was conducted using a Shimadzu LC-20AD (Shimadzu) high-performance liquid chromatography system fitted with a reversed-phase Luna C18 column and ultraviolet detection at 254 nm. Chromatographic conditions were optimised for stable retention behaviour and peak resolution. Sulfamethoxazole and trimethoprim were identified qualitatively by retention-time agreement with analytical standards. In line with the study scope, HPLC was used for identification rather than absolute quantification.

Complementary compound confirmation for atrazine was performed using gas chromatography–mass spectrometry (GC–MS) for selected extracts. Atrazine identification was based on comparison with reference mass spectral libraries and diagnostic ions. Because certified atrazine reference standards were not available, quantitative determination was not performed, and GC–MS results are reported as qualitative confirmation of presence.

### 4.9. Statistical Analysis

Data were summarised using Microsoft Excel. Exploratory spatial analyses were conducted using PAST version 4.03, while inferential statistics (one-way ANOVA and Pearson correlation analysis) were performed in STATA version 17. Statistical significance was defined as *p* < 0.05.

A formal statistical sample size calculation was not performed; sampling numbers were determined based on environmental monitoring practice and logistical feasibility for capturing spatial variability across the Harare urban water system.

## 5. Conclusions

This study provides an integrated, multi-stage observational assessment of chemical occurrence, physicochemical variability, and microbiological indicators across the Harare urban water continuum. By examining wastewater effluent from the Firle Wastewater Treatment Plant, wastewater-impacted source waters associated with Lake Chivero, treated drinking water, and downstream distribution system endpoints, this study documents the co-occurrence of pharmaceutical residues, agrochemical contaminants, physicochemical instability, and microbial regrowth indicators within an interconnected urban water system.

Physicochemical monitoring revealed elevated ammonia concentrations in source water and reduced free residual chlorine at several downstream distribution locations during the 2021 assessment. These conditions coincided with detectable heterotrophic bacterial regrowth and the intermittent presence of faecal indicators and opportunistic pathogenic bacteria at consumer endpoints, despite their absence at the treatment plant outlet during the sampled events. Taken together, these observations indicate that distribution system conditions can influence the microbiological quality of delivered drinking water, complementing the performance of the treatment stage.

Qualitative chromatographic analyses identified sulfamethoxazole and trimethoprim in wastewater-impacted matrices associated with Lake Chivero, while the same pharmaceuticals together with the agrochemical atrazine were detected in wastewater effluent from the Firle Wastewater Treatment Plant, which is hydrologically linked to Lake Chivero through the Mukuvisi River system. Although concentrations were not determined and exposure thresholds were not assessed, the environmental co-occurrence of pharmaceutical residues, agrochemical contaminants, and bacterial isolates exhibiting reduced inhibition responses under the assay conditions used highlights conditions that may be relevant to antimicrobial resistance (AMR) risk within wastewater-influenced urban water systems.

Importantly, the observational design and analytical scope of this study do not permit inference of causal mechanisms, selection processes, resistance persistence, or dissemination pathways. Genetic determinants of antimicrobial resistance were not characterised, and chemical analyses were qualitative rather than quantitative. Accordingly, the findings should be interpreted as descriptive evidence of co-occurrence and system vulnerability rather than as a direct demonstration of AMR emergence, maintenance, or selection.

Within these limitations, the results underscore the importance of viewing urban water systems as integrated systems in which wastewater management, source water protection, drinking water treatment, and distribution system integrity jointly influence water quality outcomes. From a One Health perspective, the findings highlight the need for water management strategies that extend beyond treatment plant performance alone to include the control of nutrient loading, protection of source waters, and maintenance of disinfectant residual stability within distribution networks.

Future investigations incorporating quantitative chemical analyses, molecular characterisation of antimicrobial resistance genes, and biofilm-focused sampling within distribution infrastructure will be necessary to better understand the genetic basis of the observed phenotypic responses and to clarify the mechanisms underlying the inhibition patterns observed in environmental isolates.

## Figures and Tables

**Table 1:** Summary of physicochemical parameters across sampling sites (Harare, 2021).

Sampling Site	pH	Turbidity (NTU)	Colour (Hazen)	Ammonia (mg L^−1^)	Conductivity (μS/cm)	Iron (mg L^−1^)	Aluminium (mg L^−1^)
Raw	8.0 ± 0.2	6.7 ± 6.5	43.6 ± 9.67	15 ± 3.86	188 ± 21	13 ± 9.0	0.01 ± 0.04
High	7.1 ± 0.9	2.3 ± 0.40	13 ± 8.67	10.6 ± 3.88	656 ± 48	0.1 ± 0.089	0.43 ± 0.1
Low	6.9 ± 0.08	2.1 ± 0.28	15.3 ± 9.51	4.9 ± 3.13	623 ± 47	0.14 ± 0.15	0.23 ± 0.06
Warren	7.1 ± 0.18	2.1 ± 0.32	8.8 ± 2.77	4 ± 1	616 ± 36	0.1 ± 0.07	NIL
MJ-treated	7.0 ± 0.1	2.2 ± 0.43	6.5 ± 4.46	2.8 ± 0.75	618 ± 61	0.18 ± 0.07	0.1 ± 0.08
Medium	7.1 ± 0.19	5.1 ± 2.6	8 ± 2.82	1.2 ± 1.16	640 ± 36	0.23 ± 0.12	0.2 ± 0.12
Reservoir	7.2 ± 0.1	6.9 ± 0.12	16.3 ± 2.6	2.3 ± 1.55	466 ± 18	0.05 ± 0.05	0.1 ± 0.08
WHO Guideline	6.5–8.5	≤5	≤15	≤1.5	3000	≤0.5	≤0.2

Raw—raw water from Lake Chivero; High—treated water from high density suburbs, Low—treated water from low-density suburbs, Medium—treated water from medium-density suburbs, Reservoir—treated water from reservoirs; Warren—treated water from Warren Park control point; Mj-treated—treated water from Morton Jaffray water treatment plant.

**Table 2:** Free and Total Residual Chlorine Concentrations at Selected Sampling Sites (Harare in 2021).

Parameter	MJWTP (After Chlorination)	Warren Control	Reservoirs	Medium Density	Low Density	High Density	WHO Guideline
Free residual chlorine (mg L^−1^)	0.12–0.46 0.30 ± 0.17	0.10–0.46 0.20 ± 0.17	0.00–0.11 0.02 ± 0.03	0.00–0.11 0.05 ± 0.03	0.06–0.70 0.25 ± 0.19	0.05–0.42 0.15 ± 0.11	0.20–0.50
Total residual chlorine (mg L^−1^)	0.25–0.95 0.60 ± 0.35	0.15–0.95 0.50 ± 0.35	0.01–0.21 0.09 ± 0.05	0.01–0.21 0.10 ± 0.05	0.15–1.31 0.57 ± 0.34	0.10–1.06 0.34 ± 0.29	

**Table 3:** Bacterial species with evidence of health significance related to their occurrence in treated drinking water supplies (WHO guidance).

Bacteria	Health Significance	Persistence in Water Supply	Resistance to Chlorine	Relative Infectivity
*E. coli-Enterohaemorrhagic*	High	Moderate	Low	High
*Shigella*	High	Short	Low	High
*Salmonella typhi*	High	Moderate	Low	Low
Other *salmonellae*	High	May multiply	low	Low

**Table 4:** Presence (+) or absence (−) of selected bacterial taxa at sampling sites along Harare distribution network (2021).

Sampling Site	*E. coli*	*Shigella* spp.	*Salmonella*	*Klebsiella* spp.	*Proteus* spp.
Lake Chivero	+	+	+	+	+
MJ (Treated)	−	−	−	−	−
Warren Control	−	−	−	−	−
Reservoirs	−	−	−	−	−
Medium Density (Avg)	+	−	−	−	−
High Density (Avg)	+	−	+	−	−

**Table 5:** Inhibition zone diameters (mm) produced by TMP and SMX against environmental bacterial isolates at different antibiotic concentrations.

Antibiotic	Concentration (mg mL^−1^)	*Shigella* spp. Inhibition Zone (mm)	*Escherichia coli* Inhibition Zone (mm)	*Salmonella* spp. Inhibition Zone (mm)
TMP	10	32.00 ± 1.73	0	0
TMP	5	22.00 ± 1.00	0	0
TMP	2.5	20.33 ± 0.58	0	0
TMP	1.25	22.00 ± 0.00	0	0
SMX	10	0	0	0
SMX	5	0	0	0
SMX	2.5	0	0	0
SMX	1.25	0	0	0

## Data Availability

The data presented in this study are available on request from the corresponding author. Physicochemical, microbiological, chromatographic, and antimicrobial susceptibility data generated during this study are available from the corresponding author upon request.

## Abbreviations

The following abbreviations are used in this manuscript:

Category	Abbreviation	Full Term
Institutional & Regulatory	BUSE	Bindura University of Science Education
	CLSI	Clinical and Laboratory Standards Institute
	EMA	Environmental Management Agency (Zimbabwe)
	EPA	United States Environmental Protection Agency
	ISP	International Programme in the Chemical Sciences
	MCAZ	Medicines Control Authority of Zimbabwe
	WHO	World Health Organization
Systems & Infrastructure	FWTP	Firle Wastewater Treatment Plant
	WWTP	Wastewater Treatment Plant
	MJWTP	Morton Jaffray Water Treatment Plant
	POE/POU	Point-of-Entry/Point-of-Use
	STW	Sewage Treatment Works
Analytical & Physicochemical	BDL	Below Detection Limit
	BOD_5_/COD	Biochemical Oxygen Demand (5-day)/Chemical Oxygen Demand
	DO/TDS	Dissolved Oxygen/Total Dissolved Solids
	DPD	N, N-Diethyl-p-phenylenediamine (chlorine method)
	EC	Electrical Conductivity
	GC–MS	Gas Chromatography–Mass Spectrometry
	HPLC	High-Performance Liquid Chromatography
	MSPD	Matrix Solid-Phase Dispersion
	SPE	Solid Phase Extraction
	t_r_	Retention time
	IC	Ion Chromatography
	LLE	Liquid–Liquid Extraction
	LOD/LOQ	Limit of Detection/Limit of Quantitation
	MQL	Method Quantitation Limit
	ND	Non-Detect (or Not Detected)
	NTU	Nephelometric Turbidity Units
	TA/TH	Total Alkalinity/Total Hardness
	UV	Ultraviolet
Microbiological & AMR	AMR	Antimicrobial Resistance
	CFU	Colony Forming Units
	EMB	Eosin Methylene Blue (Agar)
	H_2_S	Hydrogen Sulphide (Test)
	HPC	Heterotrophic Plate Count
	MDR	Multidrug Resistance
	AR	Antibiotic Resistance
	MAC	MacConkey (Agar)
	NA	Nutrient Agar
	TSI	Triple Sugar Iron (Test)
Chemical Targets	TMP	Trimethoprim
	SMX	Sulfamethoxazole
	PhACs	Pharmaceutical Active Compounds
Statistics	ANOVA	Analysis of Variance
	PAST	Paleontological Statistics (Software)
	STATA	Data Analysis and Statistical Software
	RSD	Relative Standard Deviation
